# Molecular Characterization of BK and JC Viruses Circulating among Potential Kidney Donors in Kuwait

**DOI:** 10.1155/2013/683464

**Published:** 2013-07-09

**Authors:** Wassim Chehadeh, Susan Silpi Kurien, Mangalathillam Raman Nampoory

**Affiliations:** ^1^Virology Unit, Department of Microbiology, Faculty of Medicine, Kuwait University, P.O. Box 24923, Safat, Jabriya 13310, Kuwait; ^2^Department of Nephrology, Hamed Al-Essa Organ Transplantation Center, Ibn Sina Hospital, P.O. Box 25427, Safat, Shuwaikh 13115, Kuwait

## Abstract

BK and JC polyomaviruses can be associated with nephropathy following renal transplantation. The aim of this study was to determine the prevalence, load, and genotypes of BK and JC viruses circulated in potential kidney donors in Kuwait. The detection of polyomavirus DNA was carried out in serum and urine samples of 165 potential kidney donors. Seventy (42%) individuals were tested positive for polyomavirus DNA, of whom 20 (12%) had detectable polyomavirus DNA in their serum samples, 40 (24%) in their urine samples, and 10 (6%) in both serum and urine samples. In the group of polyomavirus-positive patients, JC DNA could be detected in 78% of urine samples and 11% of serum samples, whereas BK DNA could be detected in 7% of urine samples and 3% of serum samples. The median polyomaviral load was low. The detected BK sequences in Kuwaiti adults formed new clusters sharing common ancestor with subgroups Ib1 and IVc, which are prevalent in Asia and Europe. Additionally, around half of the detected JCV sequences in Kuwaiti adults formed new clusters within the African subtype 3. Our results suggest high rate of polyomavirus shedding among healthy adults in Kuwait that can jeopardize their suitability for kidney donation.

## 1. Introduction

Polyomaviruses share the capacity of reactivation from latency in their host under immunosuppression. The initial manifestation of BK virus (BKV) reactivation following renal transplantation is asymptomatic viruria. This is followed by viremia and overt nephropathy, which may culminate in graft loss [1–3]. While viruria is generally considered to be insignificant, viremia and nephropathy trigger reduction in immunosuppression and antiviral therapy [4–6]. In addition to BKV, JC virus (JCV) has also been shown to be involved in the development of nephropathy [7, 8]. JCV establishes latency in the kidney and B lymphocytes, and it is associated with the development of progressive multifocal leukoencephalopathy (PML) in immunocompromised individuals, usually those with acquired immune deficiency syndrome (AIDS) [9].

The nucleotide sequences of BK and JC viruses show 75% homology [10]. Genetic mutations and rearrangement are most extensively described in the noncoding control region (NCCR) of BKV [11]. There are also reports of increased mutations of the viral capsid protein 1 (VP1) gene in patients with BK nephropathy [12–15]. Based on the VP1 gene sequence, BKV was classified into 4 major subtypes [16]. BKV subtype I is widespread in the world, subtype IV is mainly distributed in east Asia and Europe, whereas subtypes II and III are rarely detected worldwide. BKV subtype I sequences were further classified into four subgroups: Ia, Ib1, Ib2, and Ic. Subgroup Ib2 is most prevalent in Europeans and West Asians, Ia in Africans, Ic in Northeast Asians, and Ib1 in Southeast Asians. BKV subtypes IV evolved into six subgroups, IVa1, IVa2, IVb1, IVb2, IVc1, and IVc2, with each having a close relationship with a particular human population [17]. There are currently eight major genotypes of JCV, which correspond to three main areas of the world—Europe, Asia, and Africa; genotypes 1 and 4 are found in Europe, genotypes 2 and 7 are in Asia, genotypes 3 and 6 are in Africa, genotype 5 is a recombinant type between types 2b and 6, and genotype 8 is found in Papua New Guinea and the Pacific Islands [18–20]. Using 18 full sequences of JCV, studies have suggested that the virus has coevolved with human migration [21, 22].

In Kuwait, several reports have shown the role of BKV in the development of nephropathy after renal transplantation [6, 23, 24]. In addition, donor-to-recipient transmission of BKV has been previously reported [25, 26]. Moreover, early reactivation of BKV in the donor's kidney has been recently described as an important initial step in the development of BKV-associated nephropathy [27]. However, the proportion of potential kidney donors shedding BKV and JCV has not yet been investigated in Kuwait. In the current study, the prevalence and load of BKV and JCV DNA in the blood and urine samples of potential kidney donors were determined, followed by VP1 gene sequencing and phylogenetic analysis to determine the genotype of detected viral sequences.

## 2. Materials and Methods

### 2.1. Study Population

From September 2010 to August 2012, 165 potential kidney donors (95 Kuwaitis and 70 non-Kuwaiti Arabs) were enrolled at the Nephrology Unit, Hamad Al-Essa Organ Transplant Center, Ministry of Health, Kuwait. Serum and urine samples have been collected from each individual after obtaining written informed consents. The ethical permission on this research study was granted by the Ethical Decision Committee of the Research Administration, Faculty of Medicine, Kuwait University.

### 2.2. Reference Viruses

BK polyomavirus (American Type Culture Collection, ATCC VR-837, Rockville, MD, USA) and JC polyomavirus (ATCC VR-1583) were used as reference materials for the PCR assays.

### 2.3. Detection of Polyomavirus DNA by Seminested PCR

Viral DNA from serum and urine samples, and from reference strains, was extracted using the QIAmp DNA Blood Mini kit (Qiagen, Hilden, Germany). Seminested PCR was performed using previously described primers [28]. In the first PCR run, the forward BKJC1 primer (5′-AAGTCTTTAGG-GTCTTCTAC-3′) and the reverse BKJC2 primer (5′-GTGCCAACCTATGGAACAGA-3′) were used to amplify a 176-bp region from the large T antigen gene of BK and JC viruses. In the second PCR run, the internal reverse primer BKJC3 (5′-GAGTCCTGGTGGAGTTCC-3′) with the forward PEP1 primer generated a 149-bp fragment.

The first PCR run was carried out using the AmpliTaq Gold PCR Master Mix (Applied Biosystems, Foster City, CA, USA) in a total volume of 25 *μ*L containing the following: 12.5 *μ*L of Master Mix, 1.5 *μ*L of DNase-free H_2_O, 0.5 *μ*L of each external primer (10 pmol/*μ*L), and 10 *μ*L of the extracted DNA. In each analysis, negative and positive controls were set up by replacing the DNA with 5 *μ*L of distilled water or 5 *μ*L of DNA extracted from the reference polyomavirus, respectively. PCR tubes were centrifuged and the tubes were loaded into the GeneAmp PCR System 9700 (Applied Biosystems). The PCR was performed at 95°C for 9 minutes, followed by 30 cycles of amplification, consisting of the denaturation at 94°C for 60 seconds, annealing at 55°C for 60 seconds, and extension at 72°C for 60 seconds. A final elongation step was allowed at 60°C for 7 minutes followed by cooling at 4°C.

The second PCR run for polyomavirus detection was performed using 12.5 *μ*L of the Master Mix, 1.5 *μ*L of DNase-free H_2_O, 0.5 *μ*L of PEP1 primer (10 pmol/*μ*L), 0.5 *μ*L of BKJC primer (10 pmol/*μ*L), and 1 *μ*L of the first PCR product. The second PCR cycling conditions were the same as those described above for the first PCR run. The amplified PCR products were analyzed on 2% agarose gel electrophoresis, and visualized using UVP BioImaging System (Ultraviolet Products, Cambridge, UK). Image processing and analyses of DNA bands were performed using Labworks Image Acquisition and Analysis Software (Ultraviolet Products).

### 2.4. Quantitation and Differentiation of Polyomaviruses by Real-Time PCR

The forward PEP1 primer (5′-AGTCTTTAGGGTCTTCTACC-3′) and the reverse PEP2 primer (5′-GGTGCCAACCTATGGAACAG-3′) [29] were used for the common detection of BKV and JCV by a real-time PCR. The sequences of BKV- and JCV-specific internal probes [29] were modified to incorporate different fluorophores as follows: the BEP-1 probe specific for BK virus (5′-(FAM)TTTTTTGGGTGGTG‘‘T”TGAGTGTTGAGAATCTGCTGT-TGCT-3′pho) was labeled at the 5′-end with the reporter molecule 6-FAM and quenched internally at a modified ‘‘T” residue with TAMRA, with phosphate at 3′ end to prevent probe extension by the polymerase. The JEP-1 probe specific for JC virus (5′-(HEX)CTTTTTAGGTGGGG‘‘T”A-GAGTGTTGGGATCCTGTGTTTTCA-3′pho) was labeled at the 5′-end with the reporter molecule HEX and quenched internally at a modified ‘‘T” residue with TAMRA, with phosphate at 3′ end to prevent probe extension by polymerase. These probes allow the differentiation between BK and JC virus.

The real-time PCR mix was prepared by adding a MicroAmp Fast Optical 96-well reaction plate (Applied Biosystems), 12.5 *μ*L of TaqMan Universal PCR master mix (Applied Biosystems), 1.3 *μ*L of PEP1 primer (10 pmol/*μ*L), 1.3 *μ*L of PEP2 primer (10 pmol/*μ*L), 0.5 *μ*L of BEP-1 probe (10 pmol/*μ*L), 0.5 *μ*L of JEP-1 probe (10 pmol/*μ*L), 3.9 *μ*L of DNase-free water, and 5 *μ*L of DNA extract. The real-time PCR was carried out in the Applied Biosystems 7500 Fast Real-Time PCR System according to the following conditions: 50°C for 2 min, 95°C for 10 minutes, and 40 cycles (95°C for 15 sec, 60°C for 1 min). To determine the viral load, standard curves were constructed with a known copy number of the 176-bp PCR amplified fragment of the large T antigen cloned into the pGEM-T Easy Vector (Promega, Madison, WI, USA) using the PEP1 and PEP2 primers. The virus DNA concentrations in clinical specimens were determined by extrapolation from the standard curve. Deionized water (PCR grade) was used as negative control. The linear range of the BKV assay was from 1.4 × 10^2^ to 1.4 × 10^11^ copies/mL, and the linear range of the JCV assay was from 2.7 × 10^2^ to 2.7 × 10^11^ copies/mL. Both BKV and JCV real-time PCR assays were able to detect a minimum of 100 viral DNA copies/ml in the clinical specimens (serum and urine).

### 2.5. Sequencing of Detected Polyomavirus DNA

A 434-bp fragment of the polyomavirus VP1 gene that distinguishes all BK and JC genotypes [16, 18, 30] was amplified by PCR using the common primers PoE1s (5′-GGAGGAGTAGAAGTTCTAGAA-3′; nucleotides 1654 to 1674 of BKV Genbank accession number NC_001538) and PoE2as (5′-TCTGGGTACTTTGTYCTGTA-3′; nucleotides 2087 to 2067) [31].

The PCR product was purified using the Wizard SV GEL and PCR Clean-Up System kit (Promega Corporation, Madison, USA), and the nucleotide sequences of both DNA strands were then determined by direct double-strand DNA cycle sequencing with PoE1s and PoE2as primers using the ABI PRISM BigDye Terminator Cycle Sequencing v3.1 kit (Applied Biosystems). After sequencing PCR purification was performed to remove unbound fluorescent dye deoxy terminators using BigDye XTerminator Purification kit (Applied Biosystems). The samples were then denatured for 2 minutes at 95°C, immediately chilled on ice, and loaded on an ABI 3130*xl* Genetic Analyzer (Applied Biosystems). DNA sequences were then subjected to electrophoresis on a 50 cm capillary array using POP6 polymer (Applied Biosystems) as a separation medium and analyzed using the Sequencing Analysis Software v5.3.1 (Applied Biosystems). The obtained sequences were searched against the NCBI GenBank database using Basic Local Alignment Search Tool (BLAST) to confirm virus identification. BKV DNA sequences obtained in this study were deposited into the EMBL database under accession numbers HE650853, HE650856, and HE650872. The GenBank database accession numbers for JCV DNA sequences were HE648309 to HE648322, HE648324, HE652112, HE652117, HE652119, HE652120, HE652123, and HE652131.

### 2.6. Phylogenetic Analysis

BKV and JCV reference DNA sequences used to construct the phylogenetic trees were described elsewhere [18, 20, 21, 32, 33]. They were downloaded from NCBI GenBank database, and their accession numbers were included within the taxon labels in each phylogenetic tree. Viral DNA sequences of 432 bp, obtained in this study, were aligned together with their corresponding reference DNA sequences, using the ClustalW method available in the Molecular Evolutionary Genetics Analysis (MEGA) software version 4 [34]. Phylogenetic trees were reconstructed with the Bayesian Markov chain Monte Carlo (MCMC) approach implemented in BEAST v.1.7.4 program [35]. The Bayesian skyline (BSL) plot was run under relaxed uncorrelated log normal molecular clock using the best model of nucleotide substitution (HKY) as the closest related to that obtained in Modeltest [36]. Convergence of the parameters during the MCMC was inspected with Tracer v.1.5 [35] with uncertainties addressed as the 95% highest probability density intervals. Ten million chains were sufficient to achieve convergence of all parameters, with an effective sampling size (ESS > 200). The trees were sampled at each 1000 steps, resulting in a final file of 10,000 trees. These trees were summarized in a maximum a posteriori (MAP) tree using TreeAnotator v.1.7.4 program (part of the BEAST package) and visualized with FigTree v.1.4.0 program (http://beast.bio.ed.ac.uk/FigTree).

### 2.7. Statistical Analysis

The continuous variables such as age and viral load were expressed as median, and the two-tailed Mann-Whitney *U* test was used to assess the difference between two groups. All statistical analyses were performed using SPSS software version 17.0 for Windows (SPSS, Chicago, IL, USA).

## 3. Results

### 3.1. Prevalence and Load of BKV and JCV DNA in Potential Kidney Donors

A total of seventy (42%) out of 165 adults were tested positive for polyomavirus DNA by seminested PCR. The prevalence of detection of polyomavirus DNA in Kuwaitis and non-Kuwaitis was 42% and 43%, respectively. Altogether, twenty (12%) individuals had detectable polyomavirus DNA in their serum samples (viremia), 40 (24%) in their urine samples (viruria), and 10 (6%) in both serum and urine samples. The median age of polyomavirus-positive individuals was 34 years (range 20–56 years).

In order to differentiate between BKV and JCV infection and to measure the viral load, all polyomavirus-positive samples were tested by quantitative real-time PCR using virus-specific probes. Twenty six (37%) individuals had a viral load in blood and/or urine above the detection limit of the assay (100 copies/mL), with median polyomaviral DNA concentrations of 1.0 × 10^2^ copies/mL (range 1.0 × 10^2^–7.2 × 10^3^ copies/mL) and 2.9 × 10^2^ copies/mL (range, 1.0 × 10^2^–7.7 × 10^7^ copies/mL), in the serum and urine samples, respectively (*P* = 0.36). Among the 26 individuals with positive real-time PCR result, 1 (4%) had JC viremia, 22 (85%) had JC viruria, 2 (8%) had BK viruria, and 1 (4%) had BK viremia and viruria.

Due to the low viral load in most specimens, direct sequencing of the polyomavirus VP1 gene could be done for only 28 samples tested positive for polyomavirus DNA by seminested PCR. The type of infection (BK or JC) determined by BLAST analysis was identical to that obtained using real-time PCR. Overall, according to the VP1 sequencing results, 3 individuals (11%) had JC viremia, 22 (78%) had JC viruria, 2 (7%) had BK viruria, and 1 (3%) had BK viremia and viruria.

### 3.2. Phylogenetic Analysis of BKV and JCV Sequences

**Figure 1:**
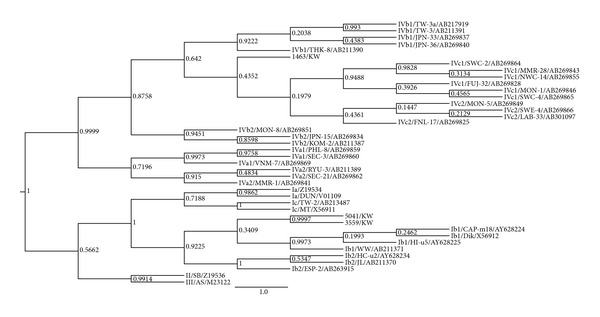
Bayesian phylogenetic analysis of a 432-bp fragment of the BKV VP1 gene. The values of posterior probability greater than 0.5 are depicted at the nodes of the trees. Each reference BKV sequence is labeled with its corresponding subtype followed by its GenBank accession number. Scale is in substitutions/site.

**Figure 2:**
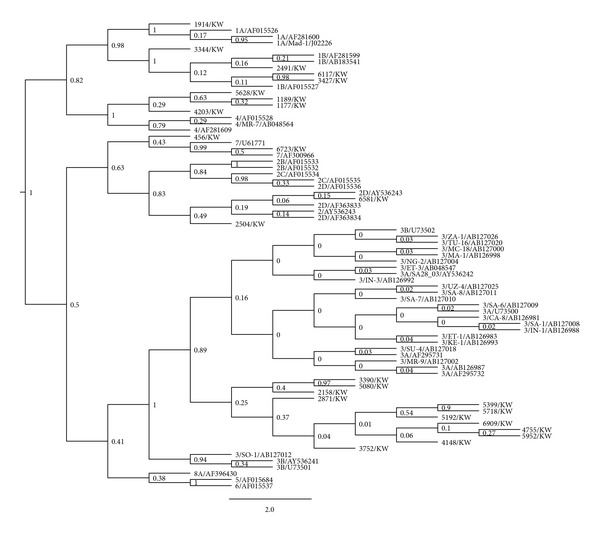
Bayesian phylogenetic analysis of a 432-bp fragment of the JCV VP1 gene. The values of posterior probability greater than 0.5 are depicted at the nodes of the trees. Each reference JCV sequence is labeled with its corresponding genotype followed by its GenBank accession number. Scale is in substitutions/site.

The VP1 sequence-based phylogenetic analysis was performed for both BKV and JCV sequences. As shown in Figure 1, BKV sequences formed new clusters in the phylogenetic tree with 2 (67%) BKV sequences sharing common ancestor with subgroup Ib1 and 1 (33%) BKV sequence sharing common ancestor with subgroup IVc. Out of 25 analyzed JCV VP1 sequences, 12 (48%) sequences formed new cluster within the subtype 3, 5 (20%) sequences were related to subtype 1, 4 (16%) to subtype 4, 2 (8%) to subtype 2, and 2 (8%) to subtype 7 (Figure 2).  Table 1 summarizes the distribution of polyomavirus genotypes among Kuwaiti and non-Kuwaiti potential kidney donors. More than half of the Kuwaiti individuals had JCV sequences with high similarity to the African subtype 3 and BKV sequences with high similarity to the Asian subtypes.

## 4. Discussion

The present study shows high rate of polyomavirus shedding by potential kidney donors. BK and JC viruses are ubiquitous human polyomaviruses that establish a latent infection. Reactivation may be brought about by immunosuppression or other factors. In a kidney transplant recipient, BKV reactivation can come from the donor or the recipient. Recipients who had BKV infection and received a kidney from the same donor have been shown to have identical BKV genotypes, supporting donor transmission [25, 26]. Recipients whose donors had higher BKV antibody titers were more likely to develop BKV infection than those with lower titers, also supporting donor transmission [25, 37]. The high rate of polyomavirus reactivation in immunocompromiszed patients is well documented, particularly in transplant recipients and AIDS patients [9, 38–40]. In renal transplantation, while 30% of patients develop asymptomatic BK viruria, only 1 to 2% develop viral nephropathy. In a previous study carried out in Kuwait, the presence of BKV in the blood of renal transplant recipients was closely correlated with the development of nephropathy [23].

The seroprevalence of BKV and JCV infections in adults ranges from 60 to 100% worldwide [41–43]. Asymptomatic urinary shedding of BKV and JCV has been reported in 7% and 19% of healthy blood donors, respectively, with median viral loads of 3.51 and 4.64 log copies/mL, respectively [43]. In the current study, polyomavirus DNA was detected in 42% of potential kidney donors, a rate that is higher than that reported earlier [38, 44]. BKV and JCV DNA could also be detected in the serum of some individuals. Since the viral load in most clinical samples was relatively low, the significance of polyomavirus identification by PCR requires evaluation in the context of the clinical and pathological findings. However, it is important to know that BKV latency in humans mostly occurs is the urogenital tract, and viral sequences can be detected in up to 50% of human kidneys [45]. Peripheral blood mononuclear cells are a second important potential site of BKV latency. In healthy individuals, rates of detection vary from 0% to 94% [46]. This wide variability in incidence in different studies is, in part, due to the extremely low viral load present in potential kidney donors. Successful detection requires sensitive assays, typically a nested PCR, with great care taken to avoid contamination [47]. Other potential variables include the presence of plasma inhibitors, the transient nature of infection, the short life span of infected mononuclear cells, and differences in susceptibility of different mononuclear fractions [47]. Other sites of viral latency have also been proposed. Thus, BKV DNA has been amplified from the brain tissue of individuals without neurologic disease [48], normal bone, and bone tumors [49]. BKV has also been cloned from liver tissue [50]. Furthermore, persistent JCV infection of kidney following primary viremia with concomitant infection of peripheral blood lymphocytes and bone marrow has been reported [51].

The human polyomavirus genome displays a great deal of sequence variation, not only between JCV and BKV but also within the various subtypes [52]. Phylogenetic analysis of the polyomavirus VP1 gene has shown that BKV isolates belonged to subtypes I and IV. Interestingly, the three BKV sequences analyzed in this study formed new clusters in the phylogeny tree, most being closely related to Ib and IVc subgroups that are prevalent in Asia and Europe [33, 53]. However, more BKV strains need to be isolated and sequenced to determine more accurately the relative frequencies of BKV subtypes in the region. It has been suggested that subtype I BKV originated in Africa and that the out-of-Africa migration of modern humans gave rise to the current distribution of the four subtype I subgroups [17]. Moreover, around half of the JCV strains detected in this study belonged to the African clade (subtype 3), and the other half was distributed between Asian and European subtypes. Other studies have shown using 18 full sequences of JCV that the virus has coevolved with human migration, supporting the anthropological evidence that all humans originated from Africa [22]. Moreover, it was demonstrated that the major African subtype diverged into two subgroups, Af2-a and Af2-b [32]. Af2-a occupies a narrow domain restricted mainly to Southern Africa, whereas Af2-b that included the three Af2-b1 to b3 subgroups had spread to whole Africa (except South Africa) and neighboring regions of Asia and Europe. Each subgroup was linked to distinct human population domains with partial overlapping at their boundaries [32]. Agostini et al. had also noted that African subtype 3 isolates were closer to types 2 and 7 than to types 1, 4, or 6 and suggested the existence of an historic Asian-African link [18].

In conclusion, the high rate of polyomavirus urinary shedding by the potential kidney donors could indicate a high exposure to the virus among the Kuwaiti population. There is a predominance of BK subtypes I and IV and JC subtype 3 in Kuwait. However, due to the limited number of BKV sequences that could be analyzed in this study, further investigations are needed to investigate the presence of different BK subtypes and subgroups in the region.

## Figures and Tables

**Table 1:** Distribution of polyomavirus genotypes among Kuwaiti and non-Kuwaiti Arabs.

	BKV genotype Total *n* = 3 (*n*, %)	JCV genotype Total *n* = 25 (*n*, %)
Kuwaiti (*n* = 21)	Ib1 (2, 67%) IVc* (1, 33%)	1 (1, 5%) 2 (2, 11%) 3 (10, 55%) 4 (3, 17%) 7 (2, 11%)
Non-Kuwaiti Arabs (*n* = 7)	—	1 (4, 57%) 3 (2, 29%) 4 (1, 14%)

*One BKV sequence defined a new branch within the subtype-IV subgroup IVc.
